# Proof-of-concept recall-by-genotype study of extremely low and high Alzheimer’s polygenic risk reveals autobiographical deficits and cingulate cortex correlates

**DOI:** 10.1186/s13195-023-01362-y

**Published:** 2023-12-12

**Authors:** Thomas Lancaster, Byron Creese, Valentina Escott-Price, Ian Driver, Georgina Menzies, Zunera Khan, Anne Corbett, Clive Ballard, Julie Williams, Kevin Murphy, Hannah Chandler

**Affiliations:** 1 Department of Psychology, University of Bath, Bath, UK; 2 School of Physics and Astronomy, Cardiff University Brain Research Imaging Centre, Cardiff University, Cardiff, UK; 3 Dementia Research Institute (UKDRI), Cardiff University, Cardiff, UK; 4 Department of Clinical and Biomedical Science, Faculty of Health and Life Sciences, University of Exeter, Exeter, UK; 5 Department of Life Sciences, Brunel University London, Uxbridge, west London UK; 6 Division of Neuroscience and Mental Health, School of Medicine, Cardiff University, Cardiff, UK; 7 School of Biosciences, Cardiff University, Cardiff, UK; 8 Institute of Psychiatry, King’s College London, Psychology & Neuroscience, London, UK; 9 Deptartment of Health & Community Sciences, University of Exeter, Exeter, UK

**Keywords:** Polygenic risk score, Alzheimer’s disease, Recall-by-genotype, Neuroimaging

## Abstract

**Background:**

Genome-wide association studies demonstrate that Alzheimer’s disease (AD) has a highly polygenic architecture, where thousands of independent genetic variants explain risk with high classification accuracy. This AD polygenic risk score (AD-PRS) has been previously linked to preclinical cognitive and neuroimaging features observed in asymptomatic individuals. However, shared variance between AD-PRS and neurocognitive features are small, suggesting limited preclinical utility.

**Methods:**

Here, we recruited sixteen clinically asymptomatic individuals (mean age 67; range 58–76) with either extremely low / high AD-PRS (defined as at least 2 standard deviations from the wider sample mean (*N* = 4504; *N*_EFFECTIVE_ = 90)) with comparable age sex and education level. We assessed group differences in autobiographical memory and T1-weighted structural neuroimaging features.

**Results:**

We observed marked reductions in autobiographical recollection (Cohen’s *d* =  − 1.66; *P*_FDR_ = 0.014) and midline structure (cingulate) thickness (Cohen’s *d* =  − 1.55, *P*_FDR_ = 0.05), with no difference in hippocampal volume (*P* > 0.3). We further confirm the negative association between AD-PRS and cingulate thickness in a larger study with a comparable age (*N* = 31,966, *β* =  − 0.002, *P* = 0.011), supporting the validity of our approach.

**Conclusions:**

These observations conform with multiple streams of prior evidence suggesting alterations in cingulate structures may occur in individuals with higher AD genetic risk. We were able to use a genetically informed research design strategy that significantly improved the efficiency and power of the study. Thus, we further demonstrate that the recall-by-genotype of AD-PRS from wider samples is a promising approach for the detection, assessment, and intervention in specific individuals with increased AD genetic risk.

## Introduction

Genome-wide associations studies (GWAS) demonstrate that genetic risk for Alzheimer’s disease (AD) can be partly explained by the cumulative impact of thousands of single-nucleotide polymorphisms (SNPs) [1, 2]. Downstream analysis of AD genetic architecture has now uncovered many novel mechanistic insights into the aetiology of AD, suggesting multiple, novel components of distinct molecular aetiology [3, 4]. Furthermore, polygenic risk scores (PRS) derived from these AD GWAS show predictive capacity to identify individuals at high risk for AD, which may provide clinical utility for early detection, intervention, and diagnosis [5–8]. The AD-PRS has been further linked to an increase across a range of preclinical AD features, including peripheral, neuroimaging, and cognitive markers of brain health, suggesting common AD genetic risk may manifest before the onset of symptoms, via alterations in neurobiological process that increase susceptibility in later life.

A significant proportion of common genetic risk for AD can be explained by the *APOE* locus, which may manifest via alterations in brain structure/function. Multiple GWAS of neuroimaging features have linked *APOE* status to amyloid burden [9], white matter hyperintensities [10, 11] and functional-temporal coherence of blood oxygen level dependency (BOLD) signals [12, 13]. Ongoing population AD-PRS studies that consider *APOE* have demonstrated associations with feature of brain health such as hippocampal volume [14]. As *APOE* status accounts for a considerable proportion of AD genetic risk, it remains largely unknown how other AD genetic risk factors link to brain structure and function. However, recent studies demonstrate an association between AD-PRS which excludes the *APOE* region (non-*APOE* AD-PRS), where non-*APOE* AD-PRS is independently linked to cognitive trajectories [15] and neuroimaging features of brain health such as hippocampal volume [16–18] and cerebrovasculature [19, 20].

However, the shared variance between AD-PRS and these preclinical features is small, limiting the utility of integrating biomarkers into prediction models of preclinical AD. One recent strategy is to assess risk in individuals with extremely high AD-PRS (defined as over two standard deviations from the sample mean). Exploring risk for AD in individuals with extreme AD-PRS values allows us to explore in vivo correlates of genetic susceptibility while limiting confounding and reverse causation that exist in samples of clinically ascertained participants [21].

Understanding non-A*POE* AD-PRS contributions to brain health remain a critical avenue of exploration when linking the common genetic architecture of AD to brain health, as to build a multiplex model of AD susceptibility and quantify downstream effects. Here, we first describe the recall-by-genotype (RbG) approach for neuroimaging non-*APOE* AD-PRS, based on the genotyping and AD-PRS estimation across a larger, population sample. We have previously demonstrated that RbG studies based on PRS with smaller number of participants can successfully reproduce observations made in larger studies. By targeting groups at the extremes of PRS, the number of participants required to provide sufficient statistical power to observed differences is drastically reduced. For example, in a recall-by-genotype study for schizophrenia PRS, we were able to observe increased prevalence of psychotic symptoms and striatal reward–linked brain activity in a sample of approximately two hundred asymptomatic participants [22], previously observed in samples of thousands [23, 24].

By assaying a non-*APOE* AD-PRS in a large, genotyped population, we can recruit a subset of individuals from the general population who have either extremely low or high AD-PRS, enriching the sample for a large amount of variation in AD-PRS. There is considerably increased AD-PRS risk (as indexed by odds ratio [OR]) in AD-RPS between the 1st and 10th decile (more than 30-fold difference in AD risk the current study, offering considerably more power (approximately fourfold increase) than an opportunistic sample (see ‘Materials and Methods’ for further details)). This study aims to understand mechanisms by which the cumulative effect of risk SNPs for AD affect the brain. We hypothesise that burden of AD risk SNPs will be related to cognitive performance and in vivo measures of brain health such as macrostructure—based on prior observations in AD, mild cognitive impairment, and at-risk populations. We expect this research will help to elucidate biological processes by which AD genetic variants may lead to reduced brain health. By identifying the preclinical features that are linked to non-*APOE* genetic risk, we hope to identify neurocognitive features that reflect AD susceptibility before the onset of symptoms. We anticipate this work may guide future detection, intervention and prevention strategies that mitigate or offset these components of adverse brain health.

## Methods and materials

### PROTECT cohort participant characteristics

Participants were recruited from the PROTECT study (www.protectstudy.org.uk; Research Ethics Committee reference number 13/LO/1578). PROTECT is a UK-based online study aimed at identifying mental health, lifestyle and genetic predictors of cognitive ageing and dementia. Participants enrol and provide written informed consent online, which includes consent for contact to participate in other research studies. Inclusion criteria for enrolling in PROTECT at the time of this study were (1) aged 50 or over; (2) access to a computer and internet; (3) no diagnosis of dementia.

### PROTECT participant genotyping procedure and quality control

Saliva samples were collected by mail and DNA was extracted by the National Institute for Health Research South London and the Maudsley National Health Service Biomedical Research Centre. Genotyping was performed via an Illumina Global Screening Array with custom content (including directly genotyped SNPs, rs429358 & rs7412, to determine *APOE* status). The initial genotyped data sample size was 4918. Genotypes underwent standard the removal with participants with low call rate (< 95%). Individuals whose sex pedigree was not congruent with genotypes were excluded. Relatedness was estimated using KING 2.2.3, followed by inclusion of individuals that contained no pairs of individuals with a first‐, second-, or third‐degree relationship (pi_hat > 0.2) [25]. SNPs with low call rate (< 95%), significant deviation from Hardy–Weinberg (*p*‐value < 1 × 10^−6^) and those with a minor allele frequency < 1% were all excluded. Principal components (PCs) were calculated for the unrelated subset of the data using EIGENSOFT 6.1.4 after pruning using a window size of 1500 bases per 150 kb / *r*^2^ = 0.2. [26, 27]. K‐means clustering was used on the first two derived PCs to define a cluster of European ancestry individuals. PCs were then recalculated for the cluster of individuals of European ancestry, with outlier individuals removed by EIGENSOFT if exceeding a sigma threshold of 30. Finally, individuals with excess heterozygosity (± 3 standard deviations) calculated using the ‘ibc’ function in plink v1.90 were excluded [28]. After individuals were excluded removing for being related, of non‐European ancestry, of mismatched sex, outliers in the PC calculation, or detected to have excess heterozygosity left a final sample size of 4504 participants.

### Alzheimer’s disease polygenic risk calculation

Polygenic score calculations were derived using training data from the International Genomics of Alzheimer’s Project (IGAP) consortium that comprises 17,008 AD cases and 37,154 control subjects [1] with PRSice v1.25 [29]. Briefly, for each participant in PROTECT, AD-PRS were calculated by summing the number of AD risk alleles present for each SNP (0, 1, or 2), weighted by the SNP’s beta coefficient for AD from the IGAP summary statistics. Our AD-PRS-based recall-by-genotype was solely based upon a standardised PRS generated from SNPs with an AD association *p*-value threshold *P* ≤ 0.5, specifically chosen as it captured the most variance in AD case / control differences in the primary AD-PRS analysis [8], using the same clumping procedure (kb = 1000, *r*^2^ = 0.2) within an *APOE* locus excluded (chr 19: kb = 44,400–46,500) SNP set, as previously employed [8].

### Recall-by-genotype and power analysis

**Fig. 1:**
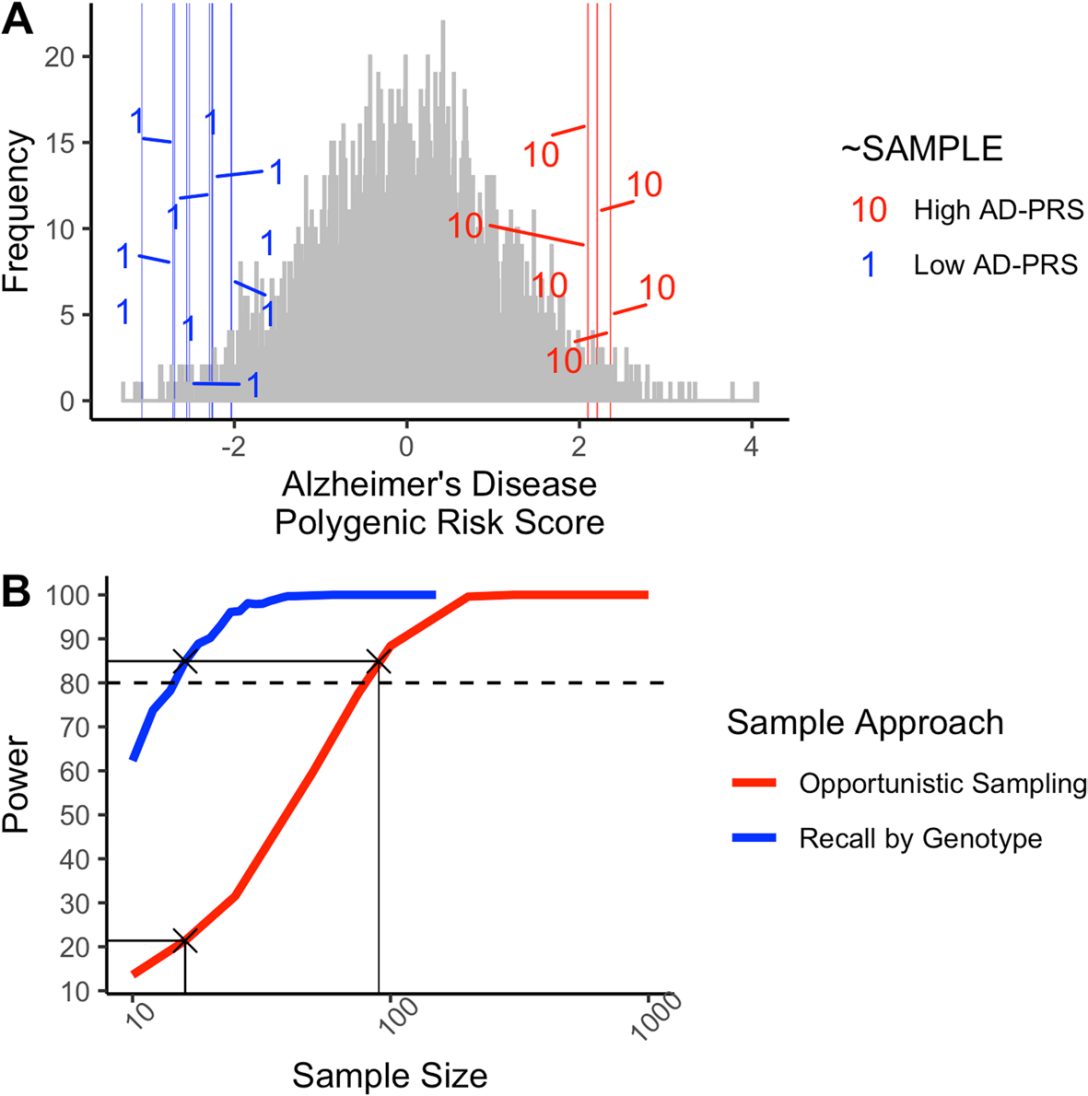
**A** Histogram represents the frequency of Alzheimer’s disease polygenic risk score (AD-PRS) estimated in 4504 participants as part of the PROTECT cohort. *X*-axis is standardised with a mean of zero and a standard deviation of 1. Grey bars reflect participants in the wider cohort, while blue and red horizontal lines represent the AD-PRS of individual participants in the present study. All participants had an AD-PRS at least 2 standard deviations over or under the cohort mean and were in the lowest (1) or highest (10) AD-PRS decile respectively. **B** Power (*y*-axis) is the proportion of significant associations from a simulated data set (from 1000 replicates), for an opportunistic sample (red line) and the recall-by-genotype sample (blue line). Crosses and black line intersections show power for our current sample (for recall-by-genotype; 85% power and opportunistic; 21% power) and the comparable sample size for the same power (*N*_EFFECTIVE_ = 90). *X*-axis reflects sample size on a log10 scale

We recruited sixteen individuals from the PROTECT cohort (*N* = 4504), who had an AD-PRS lower (*N* = 10, decile 1, blue) or higher (*N* = 6, decile 10, red) than 2 standard deviations from the population mean (Fig. 1A). Power was estimated by simulating two independent random standard normal variates *x* and *y*, and constructing a variable ‘*z* = *bY* + *x*’. Here, ‘*y*’ represents the AD-PRS, ‘*z*’ a quantitative phenotype to reflect a variable assessed via psychometric assessment or MRI, and ‘*x*’ represents the error term. The proportion of phenotype variance accounted for by the polygenic score was denoted as ‘*b*’ and was fixed at the square root of variance explained in diagnosis by AD-PRS (AUC: 0.677 / *R*^2^ = 0.095), estimated by a recent AD-PRS study for individuals under 80 years old [7]. The correlation between ‘*y*’ and ‘*z*’ is then tested in these selected samples, and power defined as the proportion of simulated samples achieving the required alpha level (*α* = 0.05). We also compare the power of the recall-by-genotype approach to an opportunistic sample of comparable sample sizes. Based on the 10 low and 6 high AD-PRS individuals we recruited from the larger PROTECT sample, we had 85% power to detect an association for the variance explained by the AD-PRS. An opportunistic sample of 90 individuals randomly sampled from this population had comparable power (Fig. 1B). By contrast, an opportunistic sample of the same size would have had 21% power. We note that this is a conservative approach to power estimation, as we could alternatively assume that considering a 30.58-fold difference in AD risk between decile 1 and 10 of AD-PRS in a comparably sized sample (*N*_GERAD (3049 cases / 1554 controls)_ = 4603 [Decile 1 vs 10]: OR = 30.58, *P* = 4.5 × 10^−87^) would yield a Cohen’s *d* = 1.875, providing for our current sample (*N* = 16) over 92% power.

### Recall-by-genotype sample characteristics

The AD-PRS groups were comparable in age (*t* =  − 1.47, *P* = 0.17), sex (*χ*^2^ = 0, *P* = 1), and *APOE* isoform status (*χ*^2^ = 2.87, *P* = 0.24) and educational attainment by highest UK qualification level [30] (*t* =  − 0.09, *P* = 0.92; *χ*^2^ = 0.178, *P* = 0.98), where the low and high AD-PRS group had a mean qualification levels of 5.1 ± 1.96 and 5 ± 2.00, respectively. Here, any potential associations between AD-PRS and behaviour / MRI features are unlikely to be explained by *APOE* ε3ε4 status as these were only present in the low AD-PRS group (Table 1).

**Table 1:** Recall-by-genotype sample descriptive statistics. Educational attainment assessed via UK guidelines based on highest level of qualification [30]

	**High (*N* = 6)**	**Low (*N* = 10)**	**Overall (*N* = 16)**
**Age at scan**			
Mean (SD)	64.3 (6.62)	69.2 (6.01)	67.4 (6.50)
Median [Min, Max]	65.0 [56.0, 72.0]	71.0 [58.0, 76.0]	68.5 [56.0, 76.0]
**Sex**			
F	4 (66.7%)	7 (70.0%)	11 (68.8%)
M	2 (33.3%)	3 (30.0%)	5 (31.3%)
***APOE* status**			
ε2ε3	1 (16.7%)	0 (0%)	1 (6.3%)
ε3ε3	5 (83.3%)	8 (80.0%)	13 (81.3%)
ε3ε4	0 (0%)	2 (20.0%)	2 (12.5%)
**Qualification level**			
Level 2	1 (16.7%)	2 (20.0%)	3 (18.8%)
Level 3	1 (16.7%)	1 (10.0%)	2 (12.5%)
Level 6	3 (50.0%)	5 (50.0%)	8 (50.0%)
Level 7	1 (16.7%)	2 (20.0%)	3 (18.8%)

The recall-by-genotype study was provided ethical approval by the Department of Psychology at Cardiff University (EC.18.12.11.5510GR2). Exclusion criteria included being older than 80 years of age, a history of psychiatric diagnosis, substance abuse, neurological disorder, or head injury; use of chemotherapy or immunomodulatory agents; genetic disorders; type I/II diabetes, cardiac, vascular, or pulmonary conditions, including a history of high blood pressure or asthma.

### Survey of autobiographical memory (SAM)

We chose to assess autobiographical memory (AM) considering the extensive and profound deficits observed in AD [31]. We assessed AM via the Survey of Autobiographical Memory (SAM) self-report instrument, assessing self-perceived AM abilities [32]. Participants completed the full-length 26-item version, rating their general memory abilities on a five-point Likert scale between strongly disagree and strongly agree. Total SAM scores as well as the four sub-domains (episodic / events, semantic, spatial, future) were calculated using the original protocol (courtesy of Brian Levine) to capture the multidimensional facets of subjective autobiographical re-experiencing.

### Structural MRI acquisition and processing

The MRI volumes were acquired on Siemens Prisma 3 T MRI scanner (*Siemens Healthineers, Erlangen, Germany*), using a 32-channel receive-only head coil. A magnetisation-prepared rapid acquisition with gradient echo (MPRAGE) T1-weighted scan was acquired (matrix 165 × 203 × 197, 1mm^3^ isotropic resolution, TR/TE = 2100/3.24 ms). Cortical and subcortical segmentations for each participant were estimated with well-validated segmentation software FreeSurfer version 7.1.1 [33], previously shown to reliably segment and parcellate grey matter tissue in AD [34]. We considered hippocampal volume (mm^3^) and cortical thickness (mm), in line with prior preclinical / AD-PRS research [14, 16–18, 35–37]. Segmentations of 66 (33 left/right) cortical grey matter regions were created based on the Desikan–Killiany atlas, and pooled to reflect bilateral macrostructural lobes (frontal, parietal, temporal, occipital and cingulate) and bilateral hippocampus volume (as well as the hemispheric total intracranial volume and average cortical thickness).

### Alzheimer’s disease polygenic risk score analysis in UK Biobank

To replicate any association between AD-PRS and MRI features, GWAS summary statistics were also acquired based on a recent MRI-GWAS in UK Biobank comprising of 33,224 individuals, which was previously corrected for demographic, neuroimaging, and genetic confounds [38]. We investigated effects of AD-PRS in the UKBB sample using the ‘gtx’ method, equivalent to the ‘inverse variance weighted’ approach in Mendelian randomisation studies [39, 40]. However, in a polygenic score analysis, there are no stringent inclusion criteria for genetic variants: we do not require the variants to be strongly associated with the outcome and pleiotropic effects are allowed. Briefly, the method uses established GWAS summary statistic data for both the exposure (AD) and outcome (GWAS summary data from MRI-image derived phenotype), which approximates the regression for an exposure (i.e. risk for AD, based on AD GWAS summary statistics) into an AD-PRS. These coefficients are weighted by SNP regression coefficients for an outcome (cortical thickness of right caudal anterior cingulate, *N* = 31,966). We used the updated AD GWAS summary statistics [41] here, which became available at time of analysis, but not before recruitment. We included SNPs at a threshold of *P*_T_ ≤ 0.5, as per our original calculation for the recall-by-genotype AD-PRS calculation [8] and removed SNPs with a minor allele frequency < 1% and imputation quality < 0.9. SNPs within both the major histocompatibility complex (chr 6: 26,000–34,000 kb) and *APOE* (chr 19: 44,400–46,500 kb) regions were also removed from the pruned dataset (*r*^2^ = 0.01, mb = 10).

## Results

### Survey of autobiographical memory (SAM)

**Fig. 2:**
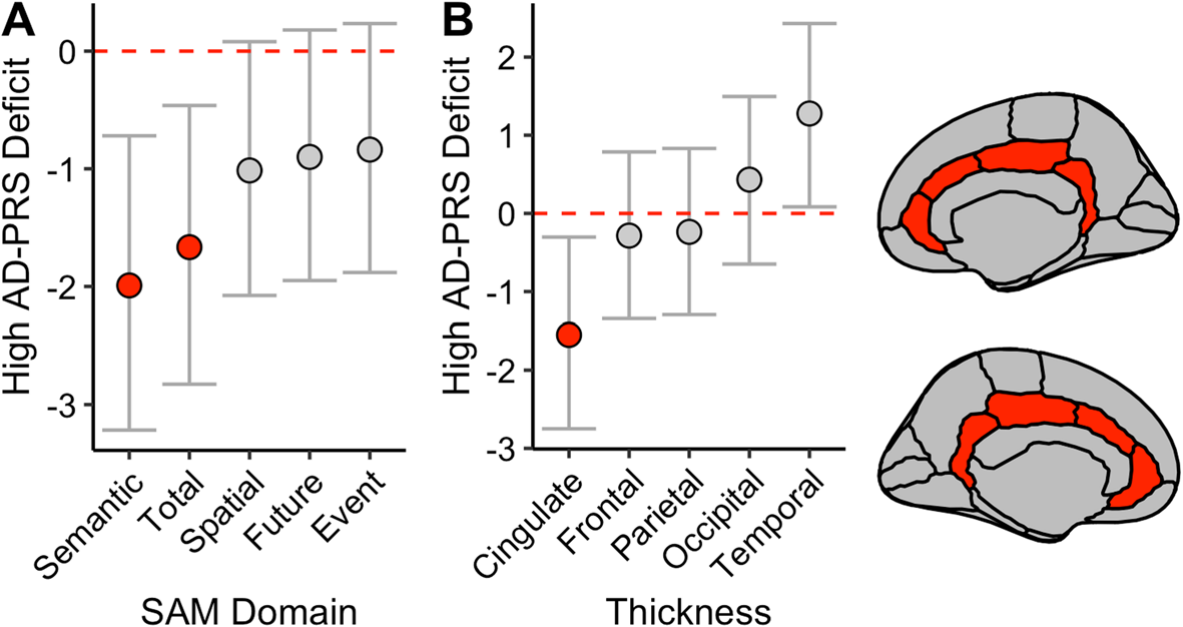
Cohen’s *d* ± 95% confidence intervals for the association between AD-PRS group and **A** SAM self-report assessment (adjusted for age and sex) and **B** average cortical thickness difference (averaged across hemisphere, adjusted for age, sex, and global cortical thickness). Significant, standardised mean differences highlighted in red; semantic, total SAM, and cingulate cortex survived correction for false discovery rate across comparisons (*P*_FDR_ < 0.05)

**Fig. 3:**
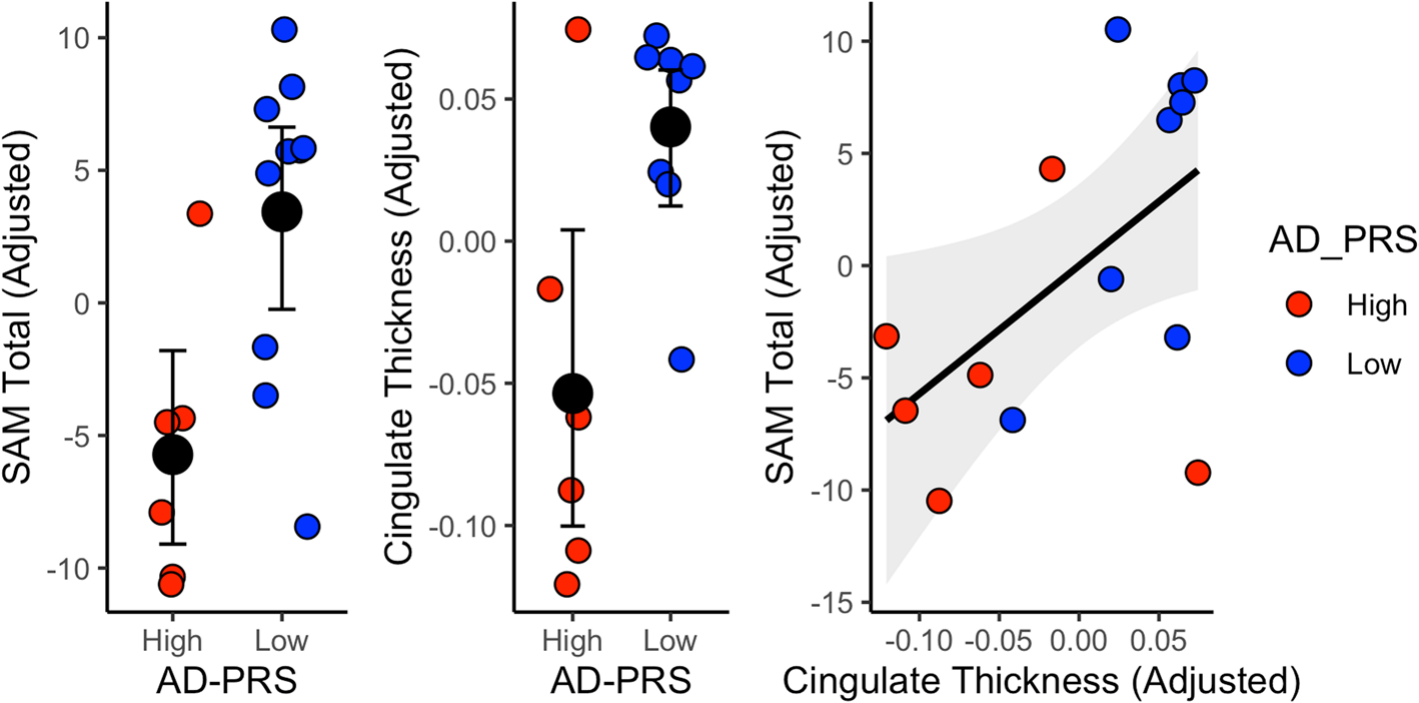
Individual data points representing AD-PRS group differences for **A** total SAM adjusted for age, sex and **B** cingulate thickness (adjusted for age, sex, and global cortical thickness). **C** Positive association between adjusted SAM total and cingulate thickness. Grey shading reflecting 95% confidence interval for line of best fit

The high AD-PRS group reported significantly reduced autobiographical memory across the total assessment (Figs. 2A and 3A: Cohen’s *d* =  − 1.66 [95%: − 2.82, − 0.46], *P*_FDR_ = 0.014). This association was further present in the sub-sample of *APOE* ε3ε3 carriers (*t* =  − 3.81, *P*_FDR_ = 0.021). A post hoc analysis demonstrated that the semantic / fact component of the assessment was most reduced in the high AD-PRS group (Cohen’s *d* =  − 1.99 [95% − 3.21, − 0.71], *P*_FDR_ = 0.006). While APOE ɛ2/3/4 status differed between the low and high AD-PRS groups (Table 1), these did not confound the AD-PRS genetic risk effects we observed, as the association was further present in the sub-sample of *APOE* ε3ε3 carriers (*N* = 13, *t* =  − 3.25, *P*_FDR_ = 0.024). No other individual components were related to AD-PRS (see Fig. 2A).

### Structural MRI

While we observed typical negative associations between hippocampal volume (mm3) and age at scan (*t* =  − 2.40, *P* = 0.039), we observed no evidence for difference in hippocampal volume between the AD-PRS groups (*P* > 0.3). The high AD-PRS group reported significantly reduced cingulate thickness (mm), corrected for age, sex, and total cortical thickness (Figs. 2B and 3B; Cohen’s *d* =  − 1.55 [95% − 2.75, − 0.30], *P*_FDR_ = 0.050). This association was further present in the sub-sample of *APOE* ε3ε3 carriers (*t* =  − 3.19, *P* = 0.019).

### Brain-behaviour associations

We further observed a positive association between total SAM and cingulate thickness, adjusted for sex, age, and total thickness (Fig. 3C; *t* = 2.36, *P* = 0.036).

### Alzheimer’s disease polygenic risk score analysis in UK Biobank

**Fig. 4:**
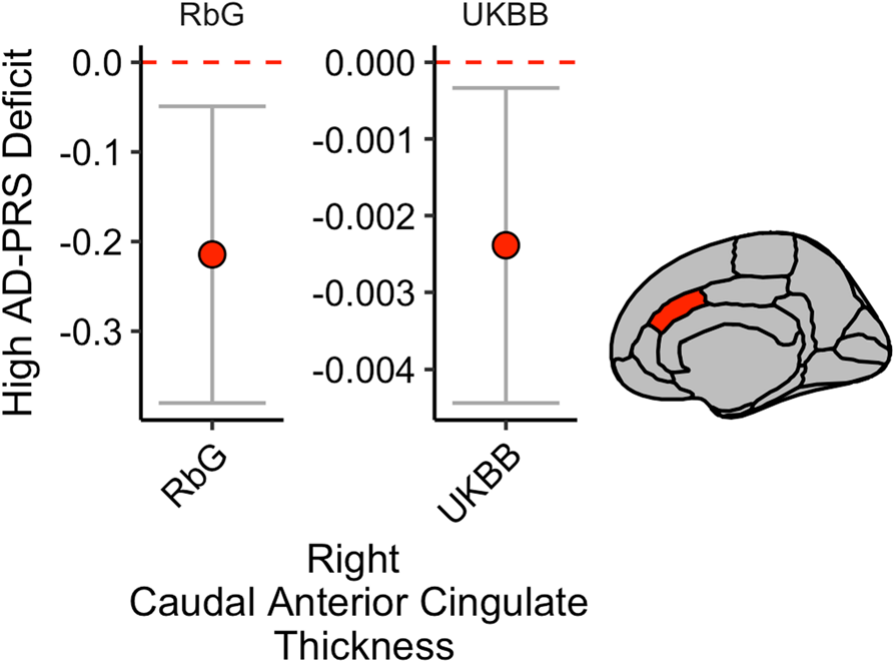
Within the cingulate cortex, the sub-region most associated was the right caudal anterior cingulate. The negative association between AD-PRS and right caudal anterior cingulate thickness in the recall-by-genotype (RbG) was replicated in the UK BioBank (UKBB) sample (*N* = 31,966). *Y*-axis represents beta estimates. Error bars represent 95% confidence intervals of the beta estimate

We observed that for the RbG sample and within the cingulate cortex, the thickness of the region cytoarchitecturally defined as the right anterior caudal cingulate cortex was most nominally associated with AD-PRS (*β* =  − 0.21 ± 0.084, *P*_UNCORRECTED_ = 0.032). We therefore acquired the summary statistics for a comparable GWAS from UK Biobank (Image Derived Phenotype ID: 1056 (aparc-Desikan_rh_thickness_caudalanteriorcingulate). We replicated this observation in this UKBB sample (*N* = 31,966; *β* =  − 0.002 ± 0.001, *P*_REPLICATION_ = 0.011) (Fig. 4).

## Discussion

We recruited sixteen individuals with either very low or high (> ± 2 SDs from population mean) to perform an assessment of autobiographical memory and T1-weighted structural MRI. Based on our estimation, an opportunistic sample (assuming a random selection of AD-PRS from the broader population) would have been significantly underpowered to detect associations with AD-PRS. However, as we were able to capitalise on variance in AD-PRS across the wider, larger sample, we were able to quadruple the power to detect AD-PRS effects, reducing the sample required from *N* = 90 to *N* = 16 [21]. We were further able to limit confounding with stringent exclusion criteria parameters.

The participants with a high AD-PRS showed marked reductions in (i) autobiographical memory and (ii) cingulate thickness compared to the low AD-PRS group. However, we did not observe an association between AD-PRS and hippocampal volume, suggesting the shared variance may be smaller than our study power afforded or that associations may be explained by confounding from genetically correlated traits such as intelligence/years of education. Several studies have demonstrated a negative association between AD-PRS hippocampal volume across the lifespan [14, 17, 35, 36]; however, a significant proportion of the shared variance has been attributed to an association with the *APOE* locus [14, 42]. Our study broadly conforms to two prior observations. First, a substantial body of evidence has previously demonstrated that increased AD genetic risk is linked to reduced cognition, with studies showing negative genetic correlations between cognition and AD common variants and AD-PRS studies supporting this observation. Second, this study supports prior observations that cortical thickness of midline / cingulate structures are reduced in individuals with high AD-PRS [37]. More specifically, reduced anterior cingulate thickness has previously been linked to AD genetic risk within a endocytosis pathway-specific AD-PRS analysis [43], across MCI groups and via associations with meta-memory in AD [44]. More recently, a negative association between AD-PRS and caudal anterior cingulate thickness has further been demonstrated in a large, pre-pubescent sample (*N*_ABCD_ > 4000), suggesting that this alteration may be a risk factor that is expressed across the lifespan [45]. While the sample age range (58–76) and autobiographical memory assessment deficit do not allow us to delineate between prodromal and early disease effects [46], we suggest that the assays we report are an initial showcase of the ability to detect AD-PRS-related differences in significantly smaller samples that could be employed at point in the lifespan, using biological readouts that are more complex and not scalable in large samples / cohorts*.* While we observed converging evidence linking AD-PRS with cingulate thickness, the study must be considered with the following limitations. While the sample size provided > 80% power to detect an association with AD-PRS, a larger or replication sample would have allowed us to further assess the validity of our findings. While this was not possible for the Survey of Autobiographical Memory (SAM), we did replicate the negative association between AD-PRS and cingulate thickness in a larger sample (*N* = 31,966). Here, we used a larger AD GWAS dataset to estimate AD-PRS [41], which became available after recruitment, an advantage of working with secondary data and updated GWAS derivates, compared to our recall-by-genotype study, which was constrained by AD-PRS estimations made before recruitment (see last section of the limitations section within this discussion). The effect size of this association was considerably smaller, this is however to be expected in a sample with additional potential sources of confounding and heterogeneity [47]. Second, we did not have a comparable group with an average AD-PRS (for example, participants with an AD-PRS in a middle decile). Therefore, any group differences we observe here may reflect higher SAM and thickness in the very low AD-PRS group, rather than preclinical alterations in the high AD-PRS group. Third, we also acknowledge that the cross-sectional design does not reveal important information such as MCI / AD conversion or trajectories which would have helped to establish the utility of the observed features in the prediction of future neurodegeneration. Fourth, while individual AD-PRS can be considered in relation to the larger sample from which they were derived, it is currently a challenge to provide an individual context about their AD-PRS as a standardised assessment. Future studies of AD-PRS working towards increased portability and generalisability, so individuals’ genetic risk can be considered independent from the sample from which their AD-PRS were estimated, may prove useful for generating AD-PRS based on existing normative samples [5, 6]. Fifth, we acknowledge that AD-PRS only represents a summated total of all known, common AD risk variants. While there are initiatives to assess AD genetic risk via the partitioning of PRS into specific biological pathways, AD-PRS may still reflect a heterogeneous, biologically unspecific estimate, making it difficult to mechanistically implicate specific causal processes. Last, we acknowledge that recall-by-genotype studies using PRS require investigators to recruit on the basis of a specific GWAS data set and with a specific PRS approach (for example, a specific P-threshold, PRS method, and GWAS training data), where variability in the process has the potential to change the position of individuals within the wider recall sample and their respective position in the AD-PRS groups, limiting their flexibility compared to compared to re-analyses of secondary data based on newer AD GWAS derivatives. While ongoing studies are working towards a standardised metric for PRS assessment [48], recall-by-genotype approaches are likely to continue being affected by ongoing GWAS and downstream methods development.

While our observations suggest that prodromal or early markers of AD pathophysiology can be observed in the high AD-PRS group, we suggest moreover that the recall-by-genotype design demonstrates that selecting specific individuals based on their PRS reflects appropriate biological features, which has several translation applications. For instance, (1) while we collected self-report memory / structural MRI features, more complex biological readouts could be assayed that are not scalable in big data such as expensive biological experiments (e.g. iPSc collection and generation) [3]; (2) genetics-first characterisation can occur across the entire lifespan, establishing precise timelines for trajectories of genetic risk, enhancing prediction, intervention opportunities, adding a layer of precision to the commonly used characteristics [49], and (3) empower clinical trials for individuals at increased genetic risk, with implications for timeliness, power, and cost [50]. Recall-by-genotype of less common, missense single-nucleotide variants (for example, functional, amino-acid change conferring SNPs within genes such as *ABI3*, *PLCG2*, *TREM2*) could also further provide mechanistic insight into the aetiology of preclinical AD, with known functional roles in modifying immune system physiology [51, 52], which have further been linked to features of brain health [53–55]. In conclusion, we document the first recall-by-genotype study for AD-PRS and observe neurocognitive features with distinct profiles between participants with very low and high AD-PRS. This recall-by-genotype approach further permits the exploration of experimental preclinical methods currently not available in large neuroimaging-genetic databases such as MRI-derived measures of cerebrovascular and neurometabolic structure and function, respectively, as well as molecular characterisation via stem cell-phenotyping [3].

## Data Availability

The wider genetic data that support the findings of this study are available from the PROTECT cohort, but restrictions apply to the availability of these data, which were used under licence for the current study, and so are not publicly available. The behaviour / MRI datasets generated and/or analysed during the current study are not publicly available as participants did not consent to public data sharing, but code supporting all inferences are available from the corresponding author on reasonable request.
